# Heterogeneous nuclear ribonucleoprotein K (hnRNP K) is a tissue biomarker for detection of early Hepatocellular carcinoma in patients with cirrhosis

**DOI:** 10.1186/1756-8722-5-37

**Published:** 2012-07-03

**Authors:** Yantong Guo, Jingming Zhao, Jingtao Bi, Quan Wu, Xin Wang, Quanyou Lai

**Affiliations:** 1 Department of Surgery, Beijing Jishuitan Hospital, the Fourth Clinical Medical College of Peking University, 31 Xinjiekou East Street, Xicheng District, Beijing, 100035, China

**Keywords:** Hepatocellular carcinoma, Proteome, Two-dimensional gel electrophoresis, Mass spectrometry, Diagnosis, Biomarker

## Abstract

**Background:**

Hepatocellular carcinoma (HCC) is one of the most common malignant tumors occurring mainly in patients with chronic liver disease. Detection of early HCC is critically important for treatment of these patients.

**Methods:**

We employed a proteomic profiling approach to identify potential biomarker for early HCC detection. Based on Barcelona Clinic Liver Cancer (BCLC) staging classification, 15 early HCC and 25 late HCC tissue samples from post-operative HCC patients and their clinicopathological data were used for the discovery of biomarkers specific for the detection of early HCC. Differential proteins among cirrhotic, early, and late tissue samples were separated by two-dimensional gel electrophoresis (2-DE) and subsequently identified by mass spectrometry (MS). Receiver operating characteristic (ROC) curves analysis were performed to find potential biomarkers associated with early HCC. Diagnosis performance of the biomarker was obtained from diagnosis test.

**Results:**

Protein spot SSP2215 was found to be significantly overexpressed in HCC, particularly in early HCC, and identified as heterogeneous nuclear ribonucleoprotein K (hnRNP K) by tandem mass spectrometry (MALDI TOF/TOF). The overexpression in HCC was subsequently validated by western blot and immunohistochemistry. ROC curve analysis showed that hnRNP K intensity could detect early HCC at 66.67 % sensitivity and 84 % specificity, which was superior to serum α-fetoprotein (AFP) in detection of early HCC. Furthermore, the diagnosis test demonstrated, when combined with hnRNP K and serum AFP as biomarker panel to detect early HCC at different cut-off value, the sensitivity and specificity could be enhanced to 93.33 % and 96 %, respectively.

**Conclusions:**

hnRNP K is a potential tissue biomarker, either alone or in combination with serum AFP, for detection of early HCC. High expression of hnRNP K could be helpful to discriminate early HCC from a nonmalignant nodule, especially for patients with liver cirrhosis.

## Findings

Hepatocellular carcinoma is one of the most common malignant tumors worldwide and is particularly prevalent in China and Asia. Persisting viral infections such as Hepatitis B (HBV) and Hepatitis C (HCV), which are the major common risk factors of HCC, is responsible for about 80% of all HCC [1]. Chronic infection with HBV in the setting of cirrhosis increases the risk of HCC 70-fold [2]. In China, most HCC cases develop in patients with advanced chronic liver disease caused by HBV infection once cirrhosis has developed, retrospective studies have suggested that patients will develop either hepatic decompensation or HCC at a rate of 2% to 7% per year [3].

For diagnosis of early HCC, patients with liver cirrhosis are advised to undergo periodic screening of serum AFP concentration and liver ultrasound at 6-to 12-monthintervals [4]. However, even with this screening strategy, many patients still present with large volume HCC (>5 cm), multifocal tumor (more than 3 lesions), or HCC that has invaded the biliary duct or portal vein. The major limitations of ultrasound is its poor ability to differentiate malignant from benign nodules in a cirrhotic liver. Serum AFP, the most commonly used biomarker of HCC, has a reported sensitivity of 39% to 65% and specificity of 65% to 94%, depend on different cut-off values. It is suggested AFP can be used to define patients at risk for HCC, but has limited utility as a screening test [5]. AFP has multiple limitations when applied to the detection of small tumors [6-8], and varies significantly in the presence of benign nodules or nonmalignant liver disease [9,10]. Therefore, there is still a need to search for biomarkers that are specifically associated with early HCC, especially in the presence of cirrhosis. Studies to extend our knowledge about the molecular pathogenesis of HCC, to identify HCC biomarkers, and therefore enable early diagnosis of HCC would be of great clinical benefit.

The proteome of tumor tissue is a rich source of cancer biomarkers, and protein released from tumor tissues may be more cancer specific than those from non-tumor tissue. Investigation of the tumor tissue proteome can identify proteomic signatures corresponding to clinicopathological features, and individual protein in such signatures may be good biomarker candidate [11]. In spite of many recent technological advances in methods for the separation and analysis of protein, two-dimensional gel electrophoresis (2-DE) coupled with tandem mass spectrometry MS is still the “gold standard” technique [12].

In the present study, proteomic 2-DE approach was used to analyze HCC patients. hnRNP K was successfully identified as a candidate biomarker for early HCC, when compared to cirrhosis controls. The sensitivity and specificity of hnRNP K alone or in combination with AFP in relevant clinical populations make this a suitable tool for the detection of early HCC.

## Materials and methods

### Patient selection

All patients included in this study suffered from cirrhosis with chronic HBV infection (Table 1). BCLC staging classification is best for treatment guidance and selection of early-stage patients that could benefit from curative therapies [13]. In this classification, early HCC is defined as a single node of HCC measuring < 3 cm, or up to 3 nodes < 3 cm each, Child-Pugh A-B class, with no symptoms and lack of change in performance status [14]. In this study, patients were divided into either early HCC group or late HCC group according to the BCLC staging system. Paired tissues were obtained from each patient, one from the cirrhotic region adjacent to the tumor and the other from the tumor region of the resected liver. The 15 recruited patients with early HCC tumors had a Child Pugh liver function score of grade A, The 25 late HCC patients were recruited with no particular selection criteria. The 40 paired cirrhotic liver tissues were diagnosed histologically, with activity ranging from mild to severe. All resected tissues were collected between 1998 and 2006 at Beijing Jishuitan Hospital. Separate tissue samples from a cohort of HCC patients (n = 20) from the same hospital were used to validate the biomarker performance. This study was approved by the ethics committee, and all the tissues were collected with informed consent from patients.

**Table 1:** Clinicopathological features of 15 early and 25 late HBV-related HCC

**Feature**	**Early HCC (n = 15)**	**Late HCC (n = 25)**
Sex		
Male	13 (87 %)	21 (84 %)
Female	2 (13 %)	4 (16 %)
Age^A^	53.67 ± 10.80	53.40 ± 12.59
AFP(ng/mL)^A^	629.07 ± 561.23	56682.04 ± 6704.74
Tumor size(cm)^A,B^	2.33 ± 0.48	10.84 ± 3.04
Venous invasion		
Absent	15 (100 %)	18 (72 %)
Present	0 (0)	7 (28 %)
Microsatlite lesion		
Absent	15 (100 %)	16 (64 %)
Present	0 (0)	9 (36 %)
Liver function		
Grade A	15 (100 %)	14 (56 %)
Grade B	0 (0)	11 (44 %)
Grade C	0 (0)	0 (0)
Tumor stage (AJCC)		
Stage I	15 (100 %)	0 (0)
Stage II	0 (0)	12 (48 %)
Stage IIIA&B	0 (0)	13 (52 %)

^A^ Mean ± SD.

^B^ Measured by the length of the largest tumor nodule.

^c^Child-pugh classification.

AFP, Alpha Fetoprotein; AJCC, AJCC, The American Joint Committee on Cancer.

### Sample preparation and 2-dimensional gel electrophoresis

Tissues were immediately snap-frozen in liquid nitrogen after surgical resection and stored at −80°C prior to analysis. Proteins were extracted from around 10 mg tissue with Bio-Rad (Hercules, CA, USA) ReadyPrepTM Sequential Extraction Kit. Tissues were first homogenized in 0.1 ml 40 mM Tris base supplemented with 20 μg/ml RNase A (bovine pancreas source, USB, Cleveland, OH, USA), 50U/ml DNase I (bovine pancreas source, USB) and complete EDTA-free protease inhibitors (Roche, Mannheim, Germany) with a mini-pestle fitted in Pellet Pestle Motor (Kontes Bineland, NJ, USA). After centrifugation at 20,800 g, pellets were homogenized in 0.1 ml extraction solution containing 8 M urea, 4%(w/v) CHAPS, 40 mM Tris, 0.2%(w/v) carrier ampholytes and 2 mM tributyl phosphine. After centrifugation at 20,800 g for 60 minutes at 15°C, protein concentrations in the supernatants were quantitated with PlusOne 2-DE Quant Kit (Amersham Biosciences, Piscataway, NJ, USA). The first-dimensional IEF was performed using precast 18 cm IPG strips at 20°C with a maximum current setting of 50 μA/strip using an AmershamBiosciences IPGphor IEF unit. IEF was carried out using the following conditions: i) 200 V, 200Vh; ii) 500 V, 500Vh; iii) 1000 V, 500Vh; iv) 1000 V-8000 V; 2250Vh; and v) 8000 V; 32000Vh. The strips were loaded onto the second dimension after equilibration. After the strips had been transferred onto 12.5% precast EttanTM DALTElectrophoresis Unit (Amersham Biosciences). Gels were then stained with a protein silver stain kit (Amersham Biosciences) following the manufacturer’s protocol.

### Gel imaging and analysis

Gel images were captured with GS-800 Calibrated Densitometer (Bio-Rad, Hercules, CA, USA). Digital images were analyzed with PDQuest 7.2 (Bio-Rad, Hercules, CA, USA). The analysis included spot detection, matching, and normalization. The intensity of each spot was normalized by total valid spot volume, and was reported as relative value (in ppm). One-way ANOVA (analysis of variance) test was used to analyze spot intensities in cirrhosis, early and late HCC samples. Only statistically significant (p < 0.01) spots were chosen for further mass spectrometric analysis.

### In-gel trypsin digestion and mass spectrometry

Protein spots were excised manually with syringe needles. Gel plugs were firstly destained with 15 mM potassium ferricyanide/50 mM sodium thiosulfate for 20 minutes and washed with Milli-Q water. The gel pieces were then equilibrated in 50 mM ammonium bicarbonate/50% methanol. After drying in SpeedVac concentrator (Savant, Farmingdale, NY, USA) for 15minutes, trypsin (MS grade, Promega, Madison, WI, USA) was added at a final concentration of 20 ng/ul to fully cover the gel pieces. Digestion was performed at 37°C overnight. Peptides were recovered with 35ul 0.1% TFA/50% acetonitrile twice. All supernatants were pooled and dried in SpeedVac concentrator for 1 hour. Prior to concentration purification with ZopTip® pipette tips (Millipore, Bedford, MA, USA), peptides were dissolved in 10ul of 0.1% TFA and followed by elution of peptides with 1 ul of 50% acetonitrile/0.1% TFA, which then mixed with same volume of matrix (10 mg/ml α-cyano-4-hydroxycinnamic acid in 50% acetonitrile 0.1% TFA). Finally, the mixtures were spotted onto the sample plate and allowed to air-dry. MS/MS spectra data were generated by ABI 4800 Proteomics Analyzer MALDI TOF/TOF (Applied Biosystems, CA, USA). A maximum of 5 most intense tandem mass spectra were collected for MS/MS in positive ion mode and of collision energy 2 kV. Mono-isotopic peaks were automatically determined and fragment ion masses were compared with the NCBI database for protein identification using the MASCOT version 2.1 (Matrix Science).

### Western blot

Twenty-five micrograms of protein extract was loaded for SDS-PAGE. After electroblotting, membrane was washed with TBS-T (20 mM Tris, 137 mM NaCl, 0.1% Tween-20, pH 7.6). The membrane was incubated in blocking solution: TBS-T/5% (w/v) non-fat dry milk/1% (w/v) BSA with gentle shaking at room temperature for 1 hour. Incubation with mouse monoclonal anti-hnRNP K antibody (Santa Cruz Biotechnology Inc., Santa Cruz, CA, USA) at 1:1500 dilution was performed at 4°C overnight. The next day, the membrane was washed with TBS-T(3 × 10 minutes), and incubated with horseradish peroxidase conjugated goat anti-mouse antibody at 1:1000 dilution for 1 hour at room temperature. After thoroughly washing with TBS-T(3 × 10 minutes), immunoreactive proteins were visualized with ECL detection reagents (Amersham Biosciences) and X-ray film (Fuji Photo Film, Tokyo, Japan). β-Actin was included as protein loading control using goat anti-human actin antibody (Santa Cruz Biotechnology, Inc.) at 1:1000 dilution for normalization.

### Immunohistochemistry

Immunohistochemistry was performed on paraffin-embedded HCC tissues at 6 μm sections that were quenched with peroxide and blocked with 3% normal goat serum and 1% BSA. Primary antibody specific for mouse anti-hnRNP K antibody (sc-28380, Santa Cruz Biotechnology Inc., Santa Cruz, CA, USA) was added to the sections and incubated at 4°C overnight, followed by the horseradish peroxidase-conjugated goat anti-mouse second antibody for 30 minutes at room temperature. The sections were observed with an Olympus light microscope using 10 × and 40× objectives.

### Statistical analysis

SPSS for Windows (version 12.0, SPSS, Chicago, IL, USA) and Graphpad Prism 5.01 was used to perform statistical analysis. One-way ANOVA, Student’s t-test were adopted in this study whenever applicable. The correlation between candidate biomarker and clinicopathological data was performed by nonparametric correlation analysis. Receiver operating characteristic (ROC) curves were performed to determine the area under the curve (AUC), and a cut-off value was selected for optimal sensitivity and specificity. The predictive performance of hnRNP K, serum AFP, were also calculated using tabular methods to evaluate the usefulness of these biomarker as individual or combined tests for early HCC according to the diagnosis test [15]. P values of less than 0.05 were considered statistically significant. Continuous data were presented as the mean ± SD.

## Results

### Overexpression of hnRNP K in early HCC tissue

**Figure 1:**
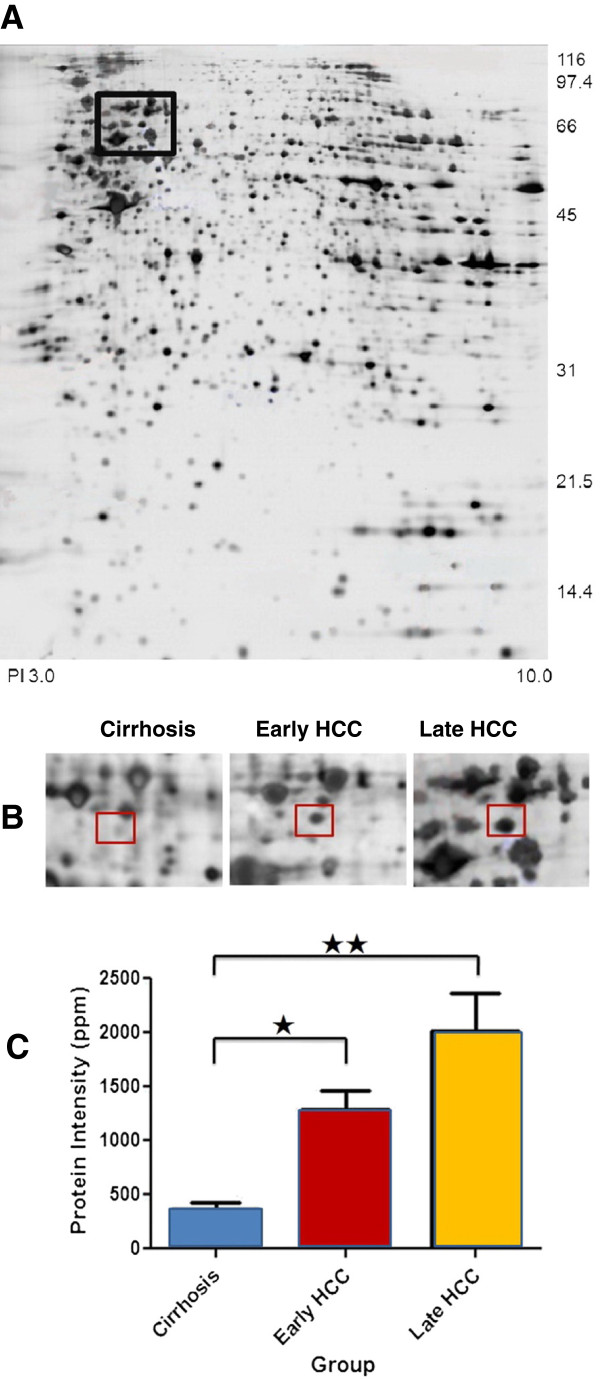
**Overexpression of hnRNP K in HCC tissues.** (**A**) Representative protein profile of tissue sample from a late HCC patient is shown. The black rectangle entails the protein spot SSP2215. (**B**) Different expression of hnRNP K among cirrhosis(n = 40), early HCC(n = 15), and late HCC(n = 25) tissues in 2-DE gel. The protein spot marked with a square on two-dimensional gel electrophoresis image is protein SSP2215. (**C**) Histograms with relative protein intensities shows protein spot SSP2215 overexpressed in early and late HCC tissues compared with cirrhosis tissue. ^☆^, p < 0.01; ^☆☆^, p < 0.01.

Protein spots that showed at least two-fold changes and a significant difference in intensity (p < 0.05) between HCC and cirrhotic liver samples were included for further analysis. Among the proteins identified as up-regulated using mass spectrometry, the protein labeled SSP2215 was found to be consistently overexpressed in HCC tissues compared with cirrhotic liver tissues (p < 0.01) (Figure 1A). This protein was expressed more strongly than other candidate biomarkers in all HCC tumors of different stages (p < 0.01) (Figure 1B). Because detection of early HCC in high risk subjects (e.g., those with cirrhosis and/or hepatitis B) could guide further treatment and improve patients’ clinical outcomes, we were motivated to distinguish any potential biomarker with expression related to early HCCs. Intriguingly, a stronger expression of SSP2215 was found to be significant in early HCCs compared to other protein spots. Moreover, significant overexpression of this protein was maintained in late HCC tumors, suggesting that expression of SSP2215 may be related to HCC development (Figure 1C). Finally, the SSP2215 spot was identified as heterogeneous nuclear ribonucleoprotein K (hnRNP K) (homo sapiens, P61978) by mass spectrometry (Table 2), and selected for further validation in a separate cohort.

**Table 2:** Summary of protein spot SSP2215 identified by tandem mass spectrometry

**Spot. No. 2215:HNRPK_Human, P61978**								
**(Heterogeneous nuclear ribonucleoprotein K hnRNP K transformation up regulated nuclear protein TUNP)**								
**Submitted mass**	**Intensity**	**Experimental mass**	**mW**	**Delta (ppm)**	**Start**	**End**	**Sequence**	**Modifications**
873.4976	19253.86	872.4898	872.4967	7.9049	397	405	(K)DLAGSIIGK(G)	-
997.4429	16550.303	996.4351	996.4335	−1.5926	317	325	(R)GGDLMAYDR(R)	-
998.3858	11027.003	997.378	997.3812	3.2434	279	286	(R)DYDDMSPR(R)	-
1013.4338	1971.4678	1012.426	1012.428	2.3512	317	325	(R)GGDLMAYDR(R)	Oxidation M (5)
1053.6353	10501.342	1052.6274	1052.634	6.3782	192	201	(R)VVLIGGKPDR(V)	-
1106.5122	32201.934	1105.5044	1105.507	2.7605	38	46	(R)NTDEMVELR(I)	-
1122.5112	3962.2905	1121.5034	1121.502	−1.0885	38	46	(R)NTDEMVELR(I)	Oxidation M (5)
1194.6987	61029.887	1193.6909	1193.692	0.8181	306	316	(R)NLPLPPPPPPR(G)	-
1259.5726	31493.924	1258.5648	1258.568	2.3278	423	433	(K)IDEPLEGSEDR(I)	-
1340.8009	16949.094	1339.7931	1339.796	2.46	208	219	(K)IILDLISESPIK(G)	-
1518.9299	20096.148	1517.9221	1517.929	4.8252	149	163	(R)LLIHQSLAGGIIGVK(G)	-
1579.7023	7277.207	1578.6945	1578.699	2.5517	22	34	(K)RPAEDMEEEQAFK(R)	-
1735.8025	8286.643	1734.7947	1734.8	2.8146	22	35	(K)RPAEDMEEEQAFKR(S)	-
1780.8018	25691.514	1779.7939	1779.791	−1.5775	70	86	(R)TDYNASVSVPDSSGPER(I)	-
1917.0393	14311.455	1916.0315	1916.026	−3.0581	378	396	(R)GSYGDLGGPIITTQVTIPK(D)	-

### Database: MASCOT, NCBInr

#### Validation of hnRNP K overexpression in HCC tissues

**Figure 2:**
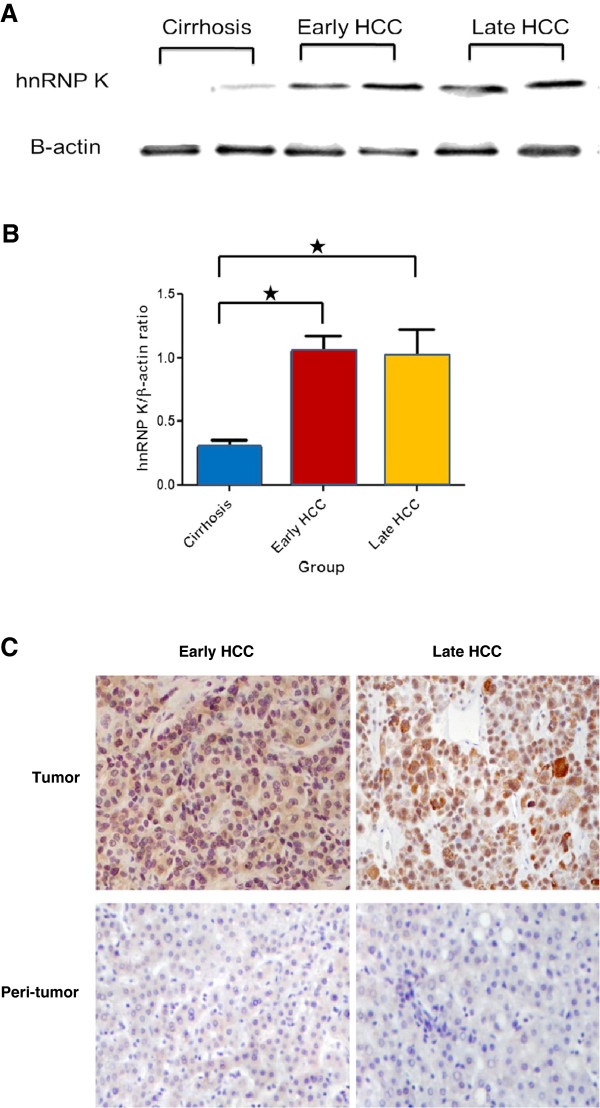
**Validation of hnRNP K overexpressed in HCC of different tumor stages.** (**A**) Representative Western blot analysis demonstrates hnRNP K overexpressed in different stages of HCC tumors compared to cirrhosis samples. β-actin was used as internal loading control. The ratio of hnRNP K/β-actin protein was measured using Scion Image Beta 4.03 software. (**B**) Histograms showed the average densitometric ratio of hnRNP K to β-actin in early and late HCC tissue compared with cirrhosis control (^☆^, p < 0.05). (Early HCC/cirrhosis = 3.48; Late HCC/cirrhosis = 3.30). (**C**) Representative Immunohistochemical staining showed the expression level and cellular localization of hnRNP K in early, late HCC tissues, and peri-tumor cirrhosis tissues. Stronger signal was found at the nucleus than that in cytoplasma in tumor cells, but limited nucleus staining in cirrhotic tissues (Original magnification × 400).

To confirm the results from 2-DE analysis, additional independent samples from 10 early HCCs, 10 late HCCs and 20 corresponding adjacent non-tumor tissues were examined to validate the hnRNP expression. Overexpression of hnRNP K protein in the HCC tissue was further confirmed by Western blot and immunohistochemistry staining. In accordance with the 2-DE results, hnRNP K expression level was higher in both the early HCC and late HCC group compared with the cirrhosis group (Figure 2). The expression of hnRNP K in the early and late HCC group was 3.5-fold and 3.3-fold higher stronger, respectively, when compared to the cirrhosis tissue (Figure 2A and 2B). Likewise, immunohistochemistry staining showed that a stronger localized immunoreactivity signal of hnRNP K was revealed in the nucleus when compared to the cytoplasm of HCC tumor cells, but limited nuclear immunoreactivity was found in cirrhotic tissues (Figure 2C).

#### Clinical significance of hnRNP K expression in HCC

Clinicopathological correlation with hnRNP K expression was analyzed in a total of 40 human HBV-associated HCCs. Overexpression of hnRNP K was shown to be significantly correlated with the increased tumor size (p = 0.022) and microsatelite nodules (p = 0.016), respectively. There was no apparent correlation with other clinicopathological features, including serum AFP concentration and venous invasion (Table 3).

**Table 3:** The correlation between hnRNP K intensity and clinicopathological variables in HCC

**1**		
**Variables**	**Coefficient**	**p value**
Serum AFP	−0.088	0.593^A^
tumor size	0.361	0.022^A^
Venous invasion	0.257	0.109^B^
Microsatellite	0.378	0.016^B^
Tumor stage(AJCC)^C^	0.322	0.059^B^

^A^ p value were obtained by Pearson correlation analysis.

^B^ p value were obtained by Spearman’s rank correlation analysis.

^C^ AJCC, The American Joint Committee on Cancer.

#### hnRNP K is a potential biomarker for early HCC

**Figure 3:**
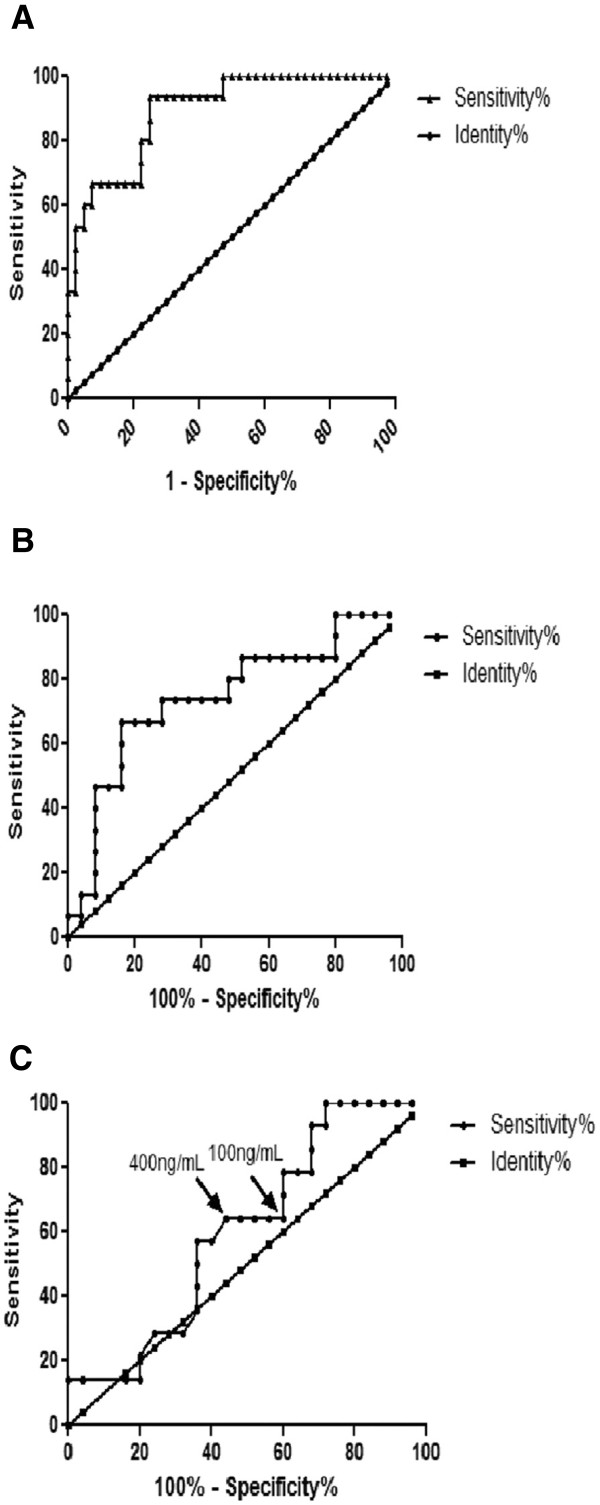
**Diagnostic performance of hnRNP K and serum AFP in detection of HCC.** The diagnostic accuracy of hnRNP K and AFP, in terms of sensitivity and specificity, are presented by receiver operating characteristic (ROC) curve analysis. In figures 3B and 3 C, corresponding to hnRNP K and AFP, the area under the curve for hnRNP K is markedly better than for the AFP in detecting early HCC. (**A**) ROC curve of hnRNP K in detection of HCC from cirrhosis (AUC = 0.89, Sen = 93.33%, Spe = 75%). (**B**) ROC curve of hnRNP K in detection of early HCC from late HCC (AUC = 0.75, Sen = 66.67%, Spe = 84%). (**C**) ROC curve of serum AFP in detection of early HCC from late HCC (AUC = 0.60). (Sen = 64.29%, Spe = 56%, cut-off value ≥ 100 ng/mL; Sen = 64.29, Spe = 40%, cut-off value ≥ 400 ng/mL). the two cut-off points of AFP are indicated with arrow.

To assess the individual performance of hnRNP K as a potential biomarker to discern early HCC from cirrhosis, and to detect early HCC from late HCC in tissue, we selected optimal fixed cutoff thresholds for hnRNP K and then calculated test sensitivity and specificity by receiver operating characteristic (ROC) curves (Figure 3). At a cutoff threshold of ≥6.396 ppm, hnRNP K showed a high accuracy to discern early HCC tissue from cirrhosis tissue with a sensitivity of 93.33% and a specificity of 75% (AUC = 0.89, p < 0.01) (Figure 3A). Likewise, hnRNP K separated early HCC from late HCC at a cutoff threshold of 7.16 ppm with a sensitivity of 66.67% and a specificity of 84% (AUC = 0.75, p < 0.01) (Figure 3B). Serum AFP had a lower diagnostic capability (AUC = 0.60, p > 0.05) of detecting early HCC from late HCC either at a cutoff value of 100 ng/mL (Sen = 64.29%, Spe = 56%) or 400 ng/mL (Sen = 64.29%, Spe = 40%) (Figure 3C), which is in accordance with the acknowledged reports that AFP is insensitive for early HCC detection.

It is well known that the combination of multiple biomarkers will improves capability for disease diagnosis. In order to further evaluate the diagnostic performance of hnRNP K with serum AFP, we performed serial and parallel diagnosis tests (Table 4). The parallel test requires that either hnRNP K or serum AFP are abnormal to classify a patient as early HCC. We used fixed cutoff thresholds of 7.16 ppm hnRNP K and 100 ng/mL AFP to discern early HCC from late HCC with a sensitivity of 93.33%, specificity of 44% and accuracy of 62.5%. In contrast, the serial test, with fixed cutoff thresholds of 7.16 ppm hnRNP K and 100 ng/mL AFP, requires that both hnRNP K and AFP are abnormal, and it increased specificity and accuracy to 96% and 72.5%, respectively, at the expense of sensitivity (33.33%) when early HCC were compared to late HCC. If a higher threshold of AFP (400 ng/mL) was combined with hnRNP K, the overall accuracy decrease either in the parallel test (62.5% vs 55%) or in the serial test (72.5% vs 70%), suggested that the combination of fixed hnRNP K with relative lower AFP (100 ng/mL) as a cutoff threshold was more powerful to diagnose early HCC.

**Table 4:** Diagnosis performance of hnRNP K and serum AFP as biomarker panel for detection of early HCC

**Test features**	**Parallel test**		**Serial test**	
	**hnRNP K ≥7.160 ppm** ***o*AFP ≥ 100 ng/mL**	**hnRNP K ≥7.160 ppm** ***or*AFP ≥ 400 ng/mL**	**hnRNP KL ≥7.160 ppm** ***and op*AFP ≥ 100 ng/mL**	**hnRNP K ≥7.160 ppm** ***and*AFP ≥ 400 ng/mL**
Sensitivity (Sen)	93.33 %	93.33 %	33.33 %	33.33 %
Specificity (Spe)	44.00 %	32.00 %	96.00 %	92.00 %
False negative rate (FNR)	0.0667	0.0667	0.6667	0.6667
False positive rate (FPR)	0.5600	0.6800	0.0400	0.0800
Youden index	0.3733	0.2533	0.2933	0.2533
Positive predictive value (PPV)	0.5000	0.4516	0.8333	0.7143
Negative predictive value (NPV)	0.9167	0.8889	0.7059	0.6970
Accuracy	0.6250	0.5500	0.7250	0.7000

## Discussion

Development of HCC is a complex process involving multiple changes in gene and protein expression. It usually occurs in the presence of liver cirrhosis. Detection of early HCC in patients with chronic liver cirrhosis is crucial, since the most effective treatments for early HCC are curative. Patients diagnosed at an early HCC stage are optimal candidates for resection, liver transplantation, or percutaneous ablation with the possibility of long term cure.

Our target was to identify disease-related proteins present in the early HCC tumor tissues by tissue proteomics, which would lead to a better understanding of the mechanisms driving tumor development. Furthermore, it has been demonstrated that protein expression profiles of cancer obtained by 2-DE and mass spectrometry could provide useful biomarkers for early detection and prognostic prediction [16,17].

Therefore, we employed 2-DE-MS proteomic analysis of 40 subjects to identify protein that enabled detection of early HCC. hnRNP K was selected to be identified and further validated both because of its high expression level in HCC tissue compared to the cirrhosis control (AUC = 0.89, p < 0.01), and its capability of distinguishing early HCC from late HCC (AUC = 0.75, p < 0.01). Validation of aberrant expression of hnRNP K in additional independent HCC tissues further reinforced the use of hnRNP K as a potential tumor marker. Correlation analysis showed that hnRNP K was not only a potential biomarker for the detection of early HCC, but also the expression level of this protein was positively correlated with the increased tumor size and the presence of microsatellites. This demonstrated that hnRNP K overexpression may be related to active tumor growth and intrahepatic micrometastasis.

hnRNP K has been shown to be involved in a number of cellular functions [18,19]. It also exhibits specific binding and transactivation within the c-myc promoter [20,21]. In addition, p53 and hnRNP K cooperate to augment transcription of target genes. Tumors simultaneously expressing p53 and hnRNP K are more aggressive [22]. In 2005, researchers identified an association between hnRNP K and the amount of HBV replication in the liver. Overexpression of hnRNP K augmented HBV replication, while gene silencing of endogenous hnRNP K carried out by small interfering RNAs resulted in a significant reduction of HBV viral load. This suggested that hnRNP K may act in concert to support the replication activity of HBV in the host [23]. In addition, hnRNP K protein promotes hepatocyte proliferation. Levels of nuclear K protein were higher in proliferating compared to resting hepatocyte [24]. It has also been demonstrated that hnRNP share three same proline-rich sequences with HCV core protein-binding domain. HCV core protein relieved the suppression effect of hnRNP K on the activity of the human thymidine kinase gene promoter[25]. These data further proved that hnRNP K may be involved in the multistep process of hepatocarcinogenesis, and eventually lead to cirrhosis and HCC by supporting HBV and HCV replication and cell proliferation.

Our verification by immunohistochemisty demonstrated that more intensive hnRNP K activity was found to localize in the nucleus compared to the cytoplasm of tumor cells, whereas faint nuclear immunostaining was observed in cirrhotic tissues. This finding is similar to a previous report that the hnRNP K expression level increased and translocated from the nucleus to the cytoplasm in colon cancer tissue [24]. The underlying molecular mechanism of hnRNP K translocation in tumor tissue need further elucidation. However, it is reasonable to infer that hnRNP K plays a different role in tumor tissue versus HBV-associated cirrhosis tissue. hnRNP K may act as a shuttle to transport important factors supporting cell growth and proliferation in tumor tissue. In HBV-associated cirrhosis tissue, hnRNP K may be involved in regulating DNA transcription to promote HBV replication. This may cause chronic liver injury, and eventually lead to the malignant transformation of the hepatocytes.

hnRNP K has been found to be overexpressed in and correlated with a poor prognosis in human prostate, lung, pancreatic, and colorectal [26-28]. The feasibility of using hnRNP K as a screening tool and a diagnostic biomarker for cancer still remains undefined. In the present study, we found that hnRNP K is overexpressed in individuals with HCC of all sizes and that it could distinguish early HCC from late HCC. This makes it a candidate biomarker for HCC screening in patients with high risk HBV infection. We compared the capability of tissue hnRNP K with serum AFP (cutoff thresholds: 100 ng/mL) in diagnosing early HCC, and as expected, hnRNP K showed a better performance than AFP in detecting early HCC (sensitivity: 66.7% vs. 64.29%, specificity: 84% vs. 56%). Because a single biomarker will not provide information regarding both tissue type and malignant transformation throughout the various stages of tumor development and progression, we further combined tissue hnRNP K intensity and serum AFP concentration to form a biomarker panel. This enhanced both the sensitivity and specificity by parallel and serial tests. The results showed that parallel test with tissue hnRNP K intensity and serum AFP cutoff thresholds optimized sensitivity (93.33%), whereas serial test optimized test specificity (96%). The combined use of hnRNP K and serum AFP has improved utility for screening and diagnosing early HCC in cirrhotic tissue.

Our study describes for the first time the usefulness of hnRNP K as a tumor biomarker for detecting early HCC, especially the detection of early HCC from liver cirrhosis. The 2-DE and immunohistochemistry data showed that hnRNP K is a specific biomarker for tumor tissue. Detecting hnRNP K expression in tissue may facilitate the accuracy of HCC diagnosis. Both the general histodiagnosis of small nodules and the distinction of high-grade dysplastic nodules form early HCC are extremely challenging, a positivity of hnRNP K staining in tissue could be taken as indicator of HCC. Despite these interesting findings, larger sample size in a randomized study is needed to fully assess the clinical value of hnRNP K. The potential application of noninvasive blood testing of hnRNP K level to detect early HCC needs to be further investigated.

## Competing interests

The authors declare that there are no conflicts of interest.

## Authors' contributions

YTG, JMZ designed the research and protocol; YTG, JTB, QW, XW and QYL were involved in collecting data and contributed to writing the manuscript. JTB, QW and XW performed the 2-dimensional gel and other laboratory assays. YTG is the principal investigator; YTG and JTB interpreted the data and wrote the paper with contributions from the other authors. All authors read and approved the final manuscript.
